# Dual-Function RNA Biomarkers: Integrating Relapse Prediction and Immune Profiling in Triple-Negative Breast Cancer

**DOI:** 10.7150/ijms.119142

**Published:** 2025-08-11

**Authors:** Ying Wen, Yuanyuan Tang, Qiongyan Zou

**Affiliations:** 1 Department of General Surgery, The Second Xiangya Hospital, Central South University, Clinical Research Center for Breast Disease in Hunan Province, No. 139, Renmin Road, Changsha, Hunan, 410011, China.; 2 Plastic surgery of breast cancer, Hunan Cancer Hospital, the Affiliated Cancer Hospital of Xiangya School of Medicine, Central South University, Changsha, Hunan, 410013, China.

**Keywords:** triple-negative breast cancer, recurrence prediction, immune infiltration, lncRNA pairs, machine learning

## Abstract

Triple-negative breast cancer (TNBC) is an aggressive breast cancer subtype with a high risk of recurrence and poor clinical outcomes. However, the factors contributing to its relapse remain inadequately understood. In this study, we utilized transcriptomic data from The Cancer Genome Atlas (TCGA) to identify lncRNA pairs associated with both recurrence and immune response. A risk prediction model was constructed through the integration of LASSO regression, Cox proportional hazards analysis, and random forest algorithms. To validate its predictive capability, we employed an external validation cohort along with a backpropagation neural network (BPNN) to assess the model's performance. Our findings indicate that the proposed risk model correlates strongly with multiple clinical features, including immune cell infiltration, response to immunotherapy, tumor mutational burden (TMB), and chemotherapy sensitivity. Additionally, a nomogram integrating risk scores with clinical parameters demonstrated superior predictive accuracy compared to models based solely on risk scores. Experimental validation confirmed that silencing LINC01605 significantly impaired TNBC cell proliferation, migration, and invasion. Overall, this risk model provides a novel approach for predicting tumor recurrence and prognosis in TNBC patients. The study also highlights the potential of LINC01605 as a therapeutic target, offering new perspectives for personalized treatment strategies.

## Introduction

Triple-negative breast cancer (TNBC) is an aggressive subtype characterized by the absence of estrogen receptor (ER), progesterone receptor (PR), and human epidermal growth factor receptor-2 (HER-2) expression 1. Due to the lack of defined molecular targets, chemotherapy remains the standard treatment, but its efficacy is limited by toxicity and drug resistance 2. Patients diagnosed with TNBC typically have a median overall survival (OS) of only 14.52 months 3, 4. Recent advances in immunotherapy, particularly immune checkpoint inhibitors (ICIs), have shown promise in improving TNBC treatment outcomes 5. Unlike other breast cancer subtypes, TNBC exhibits distinct molecular characteristics, including higher levels of tumor-infiltrating lymphocytes (TILs), which may enhance the response to immunotherapy 6. However, TNBC's heterogeneity limits ICI efficacy, underscoring the need for reliable biomarkers to predict treatment response and recurrence.

Long noncoding RNAs (lncRNAs) are emerging as critical regulators in cancer, influencing tumor progression, immune modulation, and therapy resistance 7, 8. Several lncRNA-based prognostic models have been proposed for breast cancer, such as a metabolism-related lncRNA signature for predicting recurrence-free survival (RFS) [9]and immune-related lncRNAs as potential therapeutic targets for TNBC 10. However, integrative models combining relapse prediction and immune profiling remain scarce.

Immunotherapy efficacy depends on complex interactions between tumor cells and immune modulators within the tumor microenvironment (TME), which also plays a key role in tumor recurrence 11. Some studies have demonstrated that lncRNAs are actively involved in modulating the immune landscape and influencing responses to immunotherapy 12-15. Additionally, tumor-infiltrating lymphocytes have been proposed as biomarkers for early cancer detection and immunotherapy response prediction 16. Despite these findings, there is still a lack of effective lncRNA-based models that integrate both relapse risk and immune profiling to enhance the accuracy of clinical decision-making.

In this study, we introduce a novel risk assessment model based on lncRNA pairs to predict both tumor recurrence and immune infiltration in TNBC. Unlike traditional approaches that rely on absolute gene expression levels, our innovative lncRNA pairwise binary modeling uses relative expression to create a binary matrix (e.g., 1 if lncRNA A > B, 0 otherwise), effectively minimizing batch effects and standardization biases across datasets 17. This approach enhances model robustness by reducing variability from regional, racial, or platform differences. We further strengthened the model through multi-algorithm feature selection, including LASSO regression, Cox proportional hazards analysis, and random forest, ensuring high predictive accuracy. Cross-cohort validation was performed using an external dataset and a backpropagation neural network (BPNN), which provided robust validation of the model's performance across diverse patient populations. Additionally, functional validation of the hub lncRNA LINC01605 demonstrated its role in TNBC cell proliferation, migration, and invasion, underscoring its potential as a therapeutic target. This integrative approach offers a powerful tool for prognosis prediction and personalized treatment in TNBC.

## Materials and Methods

### Data acquisition

**Figure 1:**
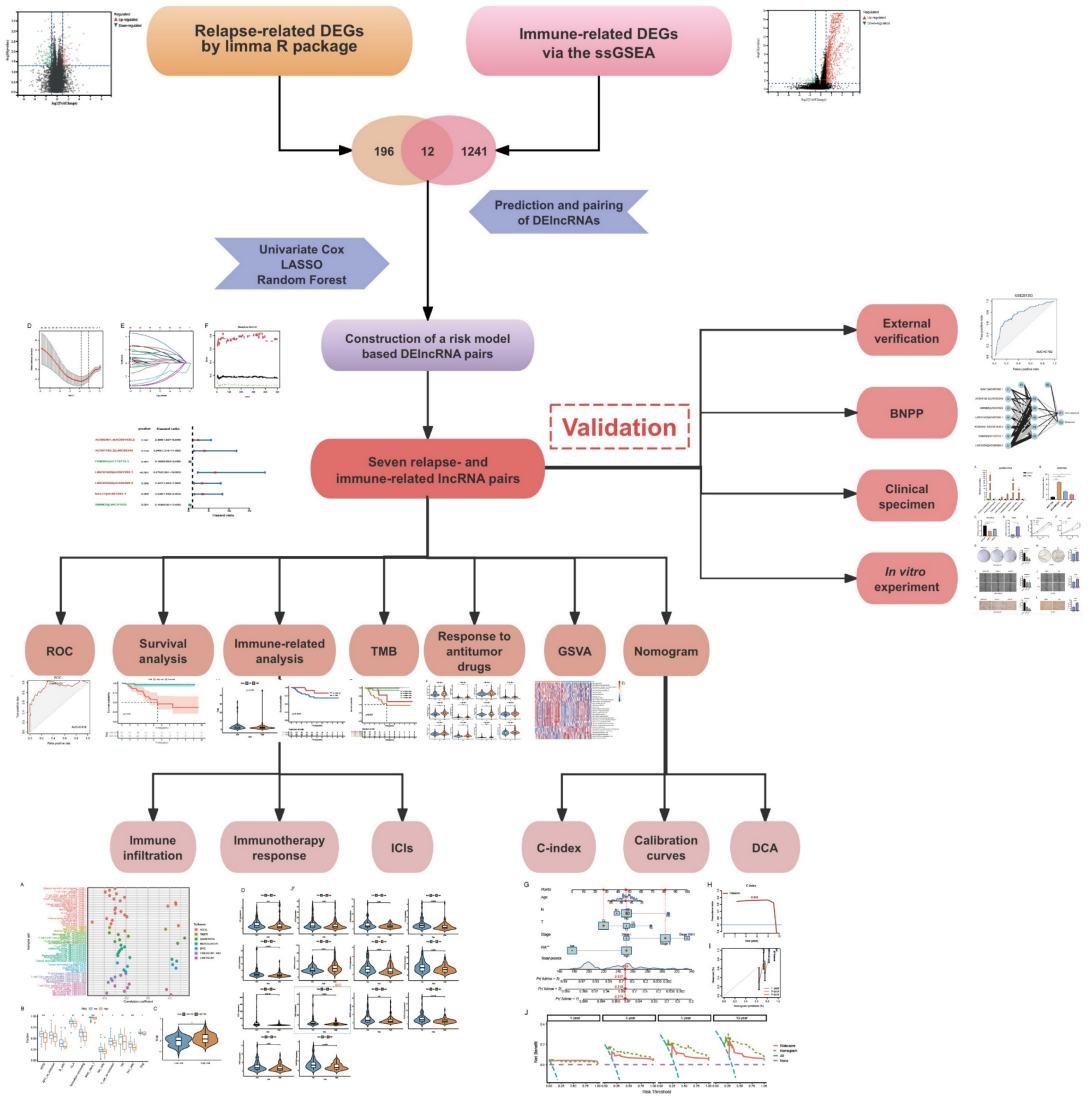
Study design and analytical workflow for identifying potential biomarkers.

We obtained transcriptome profiling data and corresponding clinical information from two publicly available databases: The Cancer Genome Atlas (TCGA) (https://portal.gdc.cancer.gov/) and the Gene Expression Omnibus (GEO) (https://www.ncbi.nlm.nih.gov/geo/). After excluding patients with incomplete clinical records, we established a cohort of 149 TNBC cases from TCGA for model construction and an external validation cohort of 106 cases from the GSE281303 dataset in GEO. The overall study design and analytical workflow are presented in **Figure 1**.

### Identification of differentially expressed relapse- and immune-related genes

We identified differentially expressed genes (DEGs) by comparing recurrence and non-recurrence patients using the limma R package, applying |log2-fold change (logFC)| > 1 and false discovery rate (FDR) < 0.05 as screening criteria. To explore the biological significance of these DEGs, we conducted gene set enrichment analysis (GSEA) using the clusterProfiler R package. To assess immune infiltration levels in each sample, we applied single-sample gene set enrichment analysis (ssGSEA). Unsupervised hierarchical clustering (K values from 2 to 10) was performed using the ConsensusClusterPlus R package to categorize samples into immune subgroups. We then identified immune-related DEGs by comparing expression levels across different immune clusters. The intersection of relapse-associated DEGs and immune-related DEGs was considered as the final set of relapse- and immune-associated genes. The clustering results and DEG distribution were visualized using the online tool Sangerbox (http://vip.sangerbox.com/home.html) 18.

### Immunophenotyping and tumor microenvironment analysis

The level of immune infiltration across different immune phenotypes was visualized using the pheatmap R package. We evaluated the tumor microenvironment (TME) using the ESTIMATE algorithm 19, which provides tumor purity, as well as immune and stromal scores. These scores were compared among different immune subgroups and illustrated using violin plots. Human leukocyte antigen (HLA), also known as the expression product of human major histocompatibility complex (MHC) glycoprotein, is closely related to the function of the human immune system 20, 21. Differences in HLA expression across immune subgroups were presented in boxplots using the ggpubr R package.

### Selection and pairing of lncRNAs

We conducted a correlation analysis between the identified relapse- and immune-related DEGs and lncRNAs to select differentially expressed lncRNAs (DElncRNAs). LncRNAs with a correlation coefficient > 0.4 and p-value < 0.001 were retained. Next, lncRNAs were paired systematically. If a pair consisted of lncRNAs A and B, we defined a new variable X such that: X = 1 if the expression of A was higher than B, X = 0 if the expression of A was lower than B. This transformation converted the expression matrix into a binary format, effectively minimizing batch effects and standardization biases introduced by regional differences, racial backgrounds, or platform inconsistencies 18, 22. This relative expression-based approach has been shown to enhance the robustness and generalizability of prognostic models across diverse datasets, as demonstrated in studies of hepatocellular carcinoma and other cancers 22. Pairs where the expression ratio was consistently 0 or 1 in over 80% of samples were excluded to ensure model stability.

### Construction of a risk assessment model

To identify lncRNA pairs associated with prognosis, we performed univariate Cox regression analysis. Candidate pairs were further refined using the least absolute shrinkage and selection operator (LASSO) regression, which was run 1,000 times using the glmnet R package. We also employed random forest (RF) analysis 23 to rank lncRNA pairs based on their importance in predicting recurrence risk. The randomForest R package was used for this step. The overlapping lncRNA pairs identified by LASSO and RF were then subjected to multivariate Cox regression to compute their coefficients (βi). The final risk score for each patient was calculated using the formula: Risk Score = ∑βi * ExpX. The predictive performance of the model was assessed using receiver operating characteristic (ROC) curves, with an optimal cutoff value determined by the survivalROC R package.

### Reverse validation by BNPP analysis

To assess the robustness of key model features, we employed a back-propagation neural network (BPNN), a widely used method in supervised learning. The BPNN consists of an input layer, one or more hidden layers, and an output layer, with interconnected neurons refining their weights through iterative learning.^24^. During training, the network processes input data, generates initial predictions, and compares them to expected outputs. The resulting error is propagated backward, adjusting synaptic weights to minimize discrepancies and improve model accuracy 25. To ensure effective model validation, TNBC patient data from the TCGA database were randomly split into two independent cohorts: 104 patients for training and 45 patients for validation, maintaining a 7:3 ratio. We employed the neuralnet, NeuralNetTools, and pROC R packages to conduct the reverse validation process. The model's predictive performance was assessed using receiver operating characteristic (ROC) curves, providing an objective evaluation of classification accuracy.

### Clinical and prognostic evaluation of the model

Kaplan‒Meier analysis was performed to compare the difference in survival in the high- or low-risk groups. The independence of the risk score as a prognostic factor was determined using univariate and multivariate Cox regression analyses. ROC curves were also plotted to compare the predictive power of the risk model with conventional clinicopathological characteristics. The boxplots demonstrated the differences in the risk score among groups sorted based on clinicopathological characteristics. A nomogram was constructed by integrating risk scores with clinical variables. The model's predictive accuracy was assessed using concordance index (C-index) and calibration curves. Additionally, decision curve analysis (DCA) was conducted using logistic regression to estimate clinical utility. The R packages were *survival*, *survminer*, *survivalROC*, *ggpubr*, *rms*, *regplot*, *pec*, and *ggDCA*.

### Immune cell infiltration and functional analysis

To explore the relationship between the risk model and immune cell infiltration, we utilized multiple computational algorithms, including XCELL, TIMER, QUANTISEQ, MCPCOUNTER, EPIC, CIBERSORT-ABS, and CIBERSORT. The correlation between the risk score and the abundance of immune cell types was visualized using a lollipop chart generated by the ggplot2 R package. Differences in immune function between high- and low-risk groups were further analyzed and illustrated in boxplots. Gene set variation analysis (GSVA) was conducted to compare functional enrichment between different risk groups. The analysis was performed using the GSVA, GSEABase, and limma R packages, with the result displayed in a heatmap.

### Association between the risk model and immune checkpoints, immunotherapy prediction response, somatic mutation and drug sensitivity

To evaluate whether the risk score is linked to immune checkpoint-related genes, we examined the expression levels of immune checkpoint inhibitors (ICIs) such as PDCD1 (PD-1), CD274 (PD-L1), CTLA-4, and LAG3. The association between the risk score and ICIs was visualized using violin plots. To predict the likelihood of response to immunotherapy, we applied the Tumor Immune Dysfunction and Exclusion (TIDE) algorithm (http://tide.dfci.harvard.edu/) 26 and compared TIDE scores between high- and low-risk groups. Higher TIDE scores indicate a greater likelihood of immune evasion and reduced sensitivity to immunotherapy. We further analyzed the correlation between risk scores and tumor mutational burden (TMB) using mutation data from TCGA-TNBC. The maftools R package was used to generate waterfall plots depicting the mutation landscape in high- and low-risk groups. Additionally, we assessed the prognostic impact of TMB by stratifying patients into high- and low-TMB subgroups. To evaluate the potential of the risk model in predicting sensitivity to chemotherapy, we estimated the half-maximal inhibitory concentration (IC_50_) values of commonly used antitumor drugs in the TCGA-TNBC cohort. Drug sensitivity predictions were made using the pRRophetic R package, and the differences in IC50 values between high- and low-risk groups were displayed using boxplots.

### Validation of key lncRNAs through clinical samples

To confirm the clinical relevance of key lncRNAs in our risk model, we collected TNBC tumor tissues and adjacent normal tissues from patients diagnosed at The Second Xiangya Hospital. Total RNA was extracted using TRIzol™ reagent (Thermo Fisher Scientific, China) and reverse-transcribed into complementary DNA (cDNA) using the Hiscript II Reverse Transcriptase Kit (Vazyme Biotech Co., Ltd.), following the manufacturer's protocol. Real-time quantitative polymerase chain reaction (RT-qPCR) was performed to quantify the expression of selected lncRNAs. The reactions were conducted using the SYBR Green qPCR Supermix kit (Invitrogen, Carlsbad, CA, USA). The amplification conditions were as follows: initial denaturation at 95°C for 3 minutes, followed by 40 cycles of denaturation at 95°C for 5 seconds and annealing/extension at 60°C for 30 seconds. The primer sequences for all target lncRNAs and the endogenous control GAPDH are provided in **Supplementary Table 1**.

### Cell culture and transfection

The human TNBC cell lines MDA-MB-231, BT549, and SUM159 were cultured in Dulbecco's Modified Eagle Medium (DMEM) (GIBCO, USA) supplemented with 10% fetal bovine serum (FBS) and 1% penicillin-streptomycin. Cells were maintained under standard conditions at 37°C in a humidified atmosphere containing 5% CO₂. Plasmids for LINC01605 overexpression and small interfering RNAs (siRNAs) targeting LINC01605 were synthesized by Tsingke (Beijing, China). Empty vectors were used as negative controls (NC). Transfection of plasmids or siRNAs was performed using Lipofectamine™ 2000 (Invitrogen, USA), following the manufacturer's protocol. The sequences of LINC01605-targeting siRNAs were as follows: siRNA-1: 5'-GAGTCTTGAAGAATAAGAAGCCACA-3'; siRNA-2: 5'-TCTTGAAGAATAAGAAGCCACAGCT-3'; siRNA-NC: 5'-GAGGTTGAATAAGAAGAACCTCACA-3'. Cells were incubated for 48 hours post-transfection, and transfection efficiency was evaluated using RT-qPCR.

### Cell counting kit-8 (CCK-8) assay

At 24 hours post-transfection, MDA-MB-231 and BT549 cells were harvested and seeded into 96-well plates at a density of 5 × 10³ cells/well. The cells were incubated for 0, 24, 48, and 72 hours. At each time point, 10% CCK-8 reagent was added to each well and incubated for 2 hours. The optical density (OD) was measured at 450 nm using a Thermo Scientific Multiskan FC microplate reader to evaluate cell viability.

### Colony formation assay

For long-term proliferation analysis, cells were seeded into 6-well plates at a density of 1 × 10³ cells/well and cultured for 1-2 weeks until visible colonies formed. The colonies were then: Fixed with 4% paraformaldehyde for 15 minutes. Stained with 0.5% crystal violet for 30 minutes at room temperature. Washed with phosphate-buffered saline (PBS) and air-dried. Colony images were captured, and the number of colonies was quantified using ImageJ software (NIH, USA).

### Wound healing assay

Cells were seeded into 6-well plates and grown to 90-100% confluence. A sterile 200-µL pipette tip was used to create a scratch in the cell monolayer. After washing with PBS to remove detached cells, serum-free DMEM was added. Wound areas were imaged at 0 and 24 hours using an Olympus inverted microscope (Japan) and quantified using ImageJ software to calculate wound closure rate. Experiments were performed in triplicate, with results expressed as mean ± standard deviation (SD). Statistical analysis was conducted using Student's t-test for normally distributed data or Wilcoxon rank-sum test for non-normal data, with P < 0.05 considered significant.

### Transwell invasion assay

Transwell chambers (8.0 µm pore size, Corning, USA) were used to assess cell invasion. Serum-free DMEM (200 µL) containing 2 × 10⁵ transfected cells was added to the upper chamber, with 500 µL DMEM supplemented with 10% FBS in the lower chamber as a chemoattractant. After 24 hours, invaded cells were fixed, stained with 0.5% crystal violet, and counted under a BX51 microscope (Olympus, Japan). Experiments were performed in triplicate, with results expressed as mean ± SD. Statistical analysis used Student's t-test or Wilcoxon rank-sum test based on data distribution, with P < 0.05 considered significant.

### Statistical analysis

All statistical analyses were conducted using R version 4.0.3 (https://www.R-project.org/) and GraphPad Prism software (version 8.0.1, La Jolla, CA, USA). For comparisons between the two groups, a t-test was performed when the data followed a normal distribution. If the data did not meet normality assumptions, a nonparametric test was applied. The Wilcoxon rank-sum test was used to assess differences in ICIs, TMB, immune infiltration, and drug sensitivity between the groups. All statistical analyses were two-sided, with *p* < 0.05 considered statistically significant. The significance levels were defined as follows: *p < 0.05, **p < 0.01, ***p < 0.001.

## Results

### GSEA of relapse- and immune-related genes in TNBC

**Figure 2:**
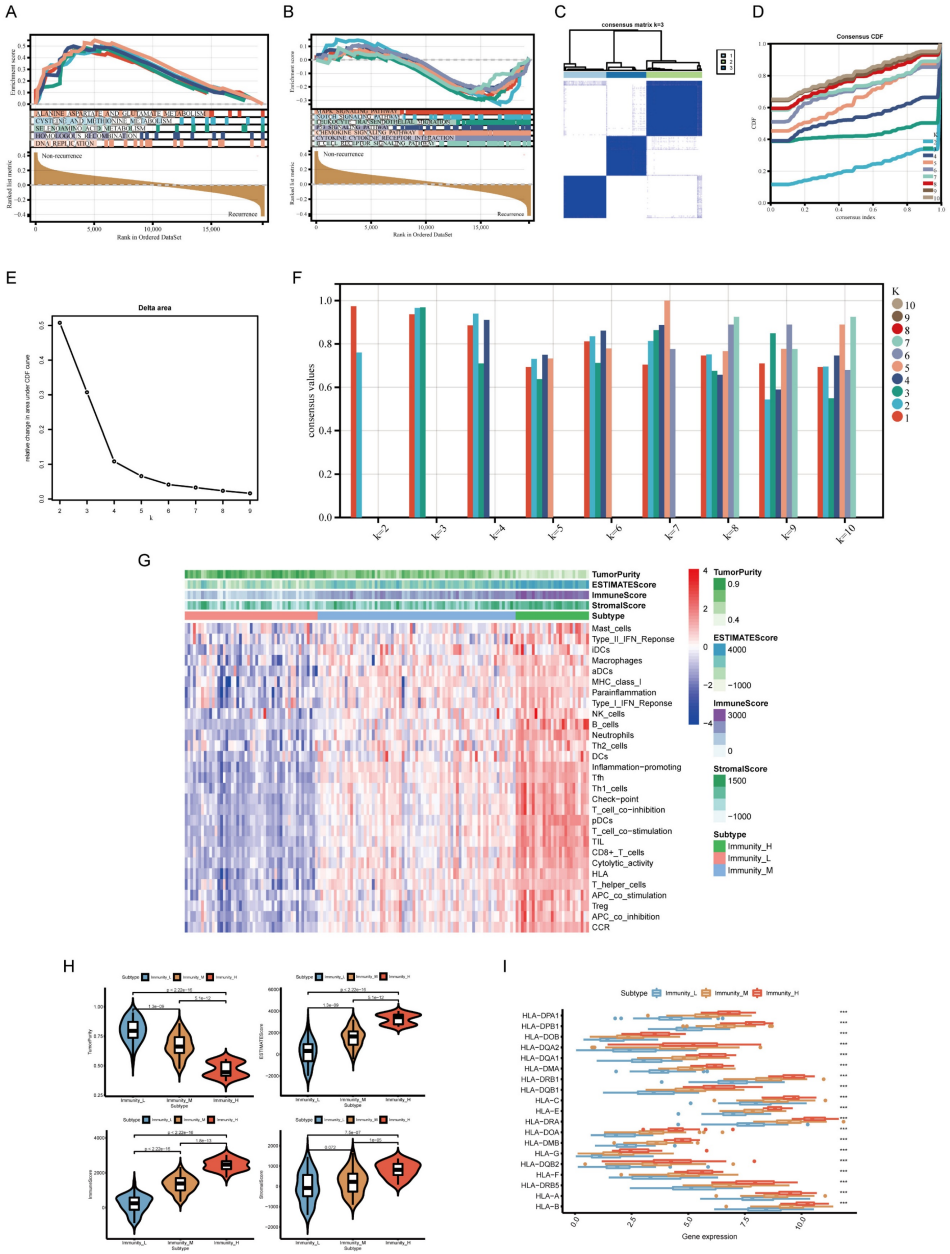
** Enrichment analysis and consensus clustering for determining the optimal K-value and validating the immune typing strategy. (A, B)** Gene Set Enrichment Analysis (GSEA) of signaling pathways enriched in non-recurrence vs. recurrence subgroups. **(C)** Consensus matrix (CM) plots at K = 3, showing optimal cluster separation. **(D)** Cumulative distribution function (CDF) plots for K-values ranging from 2 to 10. **(E)** Area under the CDF curve, indicating the stability of clustering at K = 3. **(F)** Average consistency analysis, confirming K = 3 as the most stable clustering solution. **(G)** Heatmap of tumor microenvironment (TME)-related scores, including tumor purity, ESTIMATE, immune, and stromal scores. **(H)** Comparisons of TME-related scores across different immune subgroups. **(I)** Boxplot of HLA gene expression levels across immune subgroups. ***p < 0.001.

Gene Set Enrichment Analysis (GSEA) revealed that TNBC recurrence was associated with DNA replication, homologous recombination, and amino acid metabolism pathways, while immune-related pathways such as the MAPK, NOTCH, P53, leukocyte transendothelial migration, chemokine, cytokine‒cytokine receptor interaction and B-cell receptor signaling pathways were negatively enriched in recurrent tumors (**Figure 2A, B**). These findings suggest that tumor recurrence is most likely associated with some carcinogenic pathways and immune cell functions.

### Immune subgroup classification and tumor microenvironment analysis

We performed unsupervised hierarchical clustering to determine the optimal number of clusters (K). The consensus matrix (CM) plots (**Figure 2C**) illustrate clustering patterns across different K values, with the most distinct and least noisy separation observed at K = 3, as indicated by the darkest blue squares. The cumulative distribution function (CDF) plots (**Figure 2D**) show the cumulative consensus distributions for various K values, where the slope of the CDF curve gradually stabilizes at K = 3, suggesting an optimal clustering resolution. Additionally, the relative change in the area under the CDF curve (**Figure 2E**) further supports K = 3 as a stable clustering choice. Lastly, the average consistency evaluation plot (**Figure 2F**) confirms that the highest within-group consistency is achieved at K = 3, reinforcing its suitability for sample classification.

Analysis using the ESTIMATE algorithm demonstrated that Immunity-H patients exhibited significantly higher immune and stromal scores, while Immunity-L patients had the lowest immune infiltration and highest tumor purity (**Figure 2G, H**). Additionally, human leukocyte antigen (HLA) gene expression levels were significantly elevated in the Immunity-H group compared to the Immunity-L group (**Figure 2I**), further supporting the validity of our immunophenotyping strategy.

### Construction and evaluation of the lncRNA pairs-based risk model

**Figure 3:**
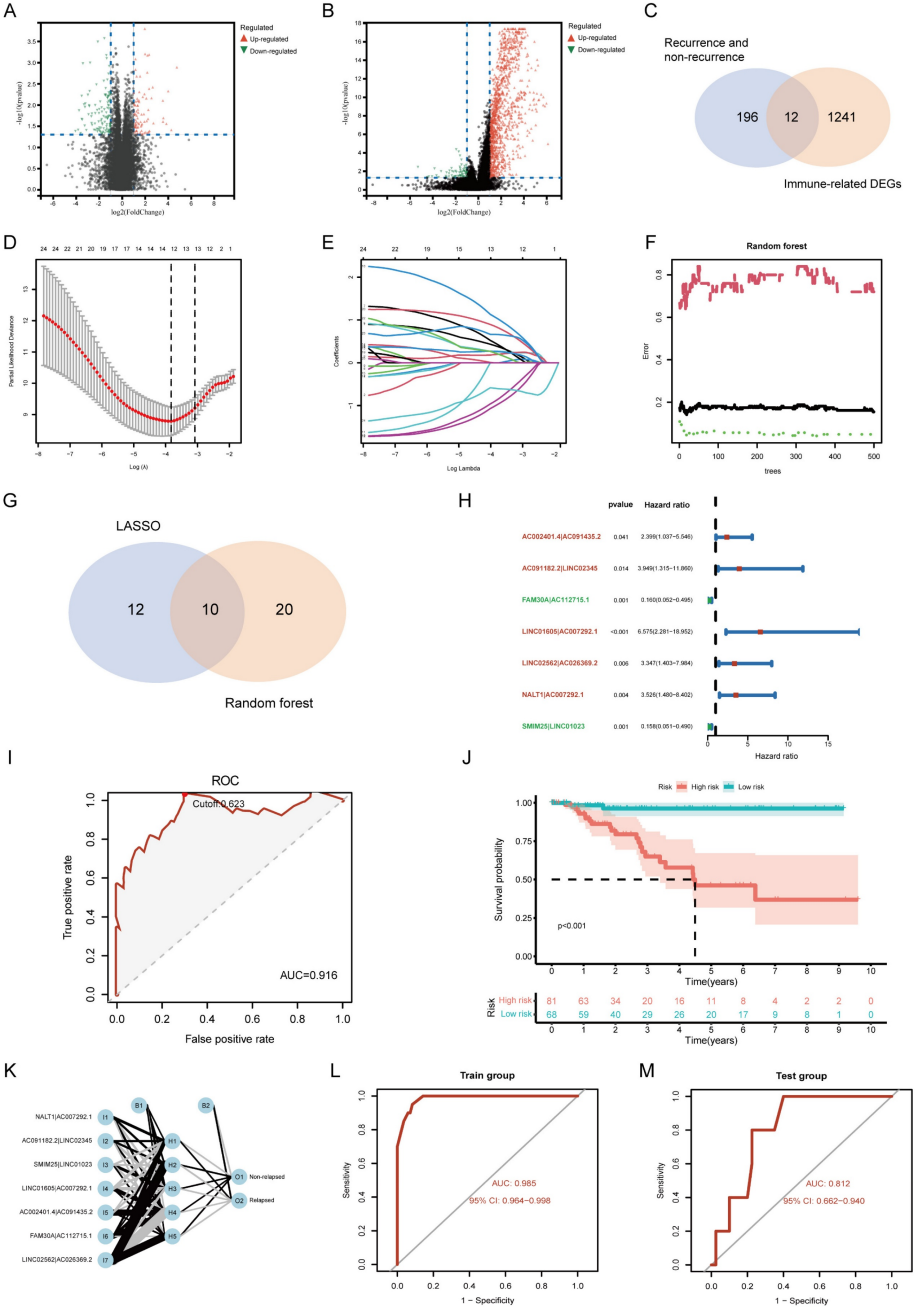
**Construction and validation of a risk assessment model based on lncRNA pairs. (A, B)** Volcano plots of differentially expressed genes (DEGs) related to recurrence and immune response.** (C)** Venn diagram showing the intersection of relapse- and immune-related DEGs.** (D, E)** Feature selection of lncRNA pairs using LASSO regression.** (F)** Random forest (RF) error rate analysis, with error rates for relapsed and non-relapsed groups shown in red and green, respectively. The overall model error rate is shown in black.** (G)** Selection of 10 lncRNA pairs identified by LASSO and RF algorithms.** (H)** Final risk assessment model comprising 7 key lncRNA pairs.** (I)** Receiver Operating Characteristic (ROC) curve, showing the optimal cutoff value determined at the maximum inflection point.** (J)** Kaplan-Meier survival curves comparing overall survival between high-risk and low-risk groups.** (K)** Validation of key model features using a backpropagation neural network (BPNN).** (L, M)** ROC curves for training and test sets, demonstrating the model's classification accuracy.

To explore potential molecular factors associated with tumor recurrence and immune response in TNBC, we analyzed transcriptomic data from the TCGA-TNBC cohort. A total of 196 differentially expressed genes (DEGs) were identified between recurrence and non-recurrence groups (|log₂FC| > 1, FDR < 0.05), while 1,241 immune-related DEGs were identified through immune subgroup clustering (**Figure 3A, B**). By intersecting these datasets, 12 key genes were found to be associated with both tumor recurrence and immune response (**Figure 3C**). Based on the identified relapse- and immune-associated genes, we constructed 6,390 lncRNA pairs using a relative expression-based pairing strategy. After filtering for prognostic relevance, 25 lncRNA pairs were selected through univariate Cox regression analysis (*p* < 0.005, **Supplementary Table 2**). Subsequently, LASSO further confirmed that 12 pairs were related to prognosis (**Figure 3D, E**), and random forest analysis indicated that 20 pairs were related to relapse risk (**Figure 3F**). **Figure 3G** displays 10 overlapping pairs, out of which 7 were selected as the basis of the risk model after performing multivariate Cox regression (**Figure 3H, Supplementary Table 3**). The model's predictive performance was assessed using ROC curve analysis, yielding an area under the curve (AUC) of 0.916, confirming strong prognostic accuracy (**Figure 3I**). It is worth noting that patients with high-risk scores exhibited a significantly poorer prognosis in comparison to those with low-risk scores (*p* < 0.001, **Figure 3J**). Using the established risk score formula, the model was further validated in the GSE281303 dataset, where similar predictive performance was observed (AUC = 0.782, **Supplementary Figure 1**). Furthermore, to enhance confidence in our findings, we employed the BPNN for independent validation. The BPNN was configured with 2 hidden layers, 5 nodes, and the output of the algorithm was whether the patient would experience a relapse (**Figure 3K**). The BPNN was trained using a 7:3 split of TCGA-TNBC patients (104 training, 45 validation). The model demonstrated excellent classification performance, with AUC values of 0.985 and 0.812 in the training and validation sets, respectively (**Figure 3L, M**).

### Assessment of the independent prognostic value and clinical evaluation of the risk model

**Figure 4:**
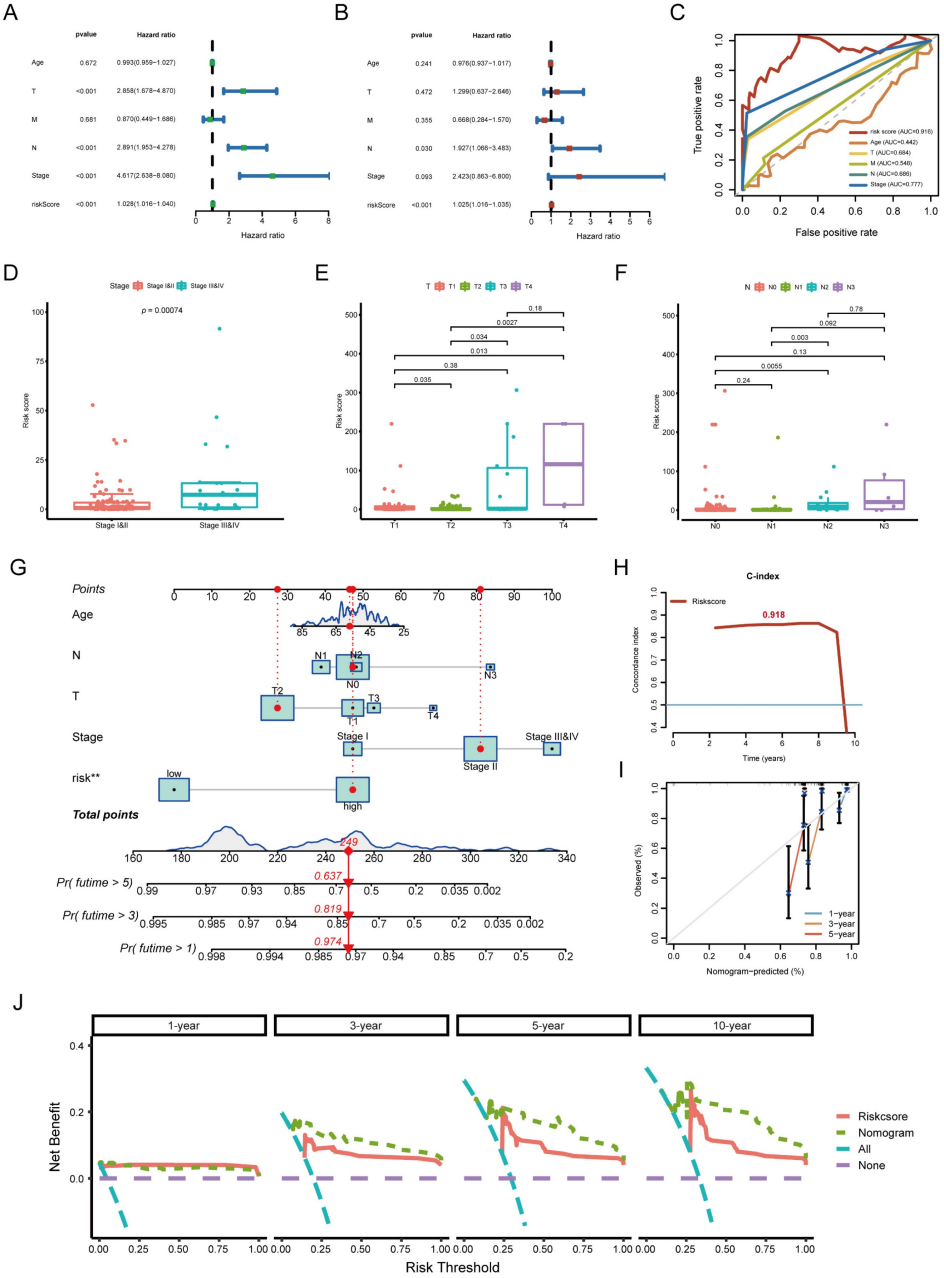
** Clinical evaluation of the lncRNA pair-based risk model. (A, B)** Forest plots from univariate and multivariate Cox regression analyses, demonstrating the prognostic significance of the risk score. **(C)** Comparison of ROC curves between the risk model and clinicopathological features, confirming the superior predictive performance of the risk score. **(D, E, F)** Association between risk score and clinical parameters, including clinical stage, T stage, and N stage. **(G)** Nomogram for predicting 1-, 3-, and 5-year survival rates in TNBC patients. **(H, I)** C-index and calibration curves, assessing the accuracy and calibration of the nomogram. **(J)** Decision curve analysis (DCA), comparing the clinical benefit of different models: Nomogram, Risk score, “All” strategy, and “None” strategy.

To determine whether the risk model provided independent prognostic value, we performed univariate and multivariate Cox regression analyses. The risk score remained a significant independent predictor of TNBC prognosis, even after adjusting for clinicopathological characteristics (**Figure 4A, B**). Moreover, the risk score outperformed traditional clinicopathological features in ROC curve comparisons, confirming its superior predictive power (**Figure 4C**). Boxplot analysis showed that higher risk scores were significantly associated with advanced clinical stage, larger tumor size (T stage), and lymph node involvement (N stage) (**Figure 4D-F**). These findings highlight the clinical relevance of the risk model in stratifying TNBC patients. A nomogram integrating the risk score with clinical parameters was constructed to improve individual patient risk assessment. The C-index (0.918) indicated strong predictive capability, and calibration curves demonstrated high agreement between predicted and actual survival probabilities (**Figure 4G-I**). Decision curve analysis (DCA) further confirmed that the nomogram provided greater clinical benefits than the risk score alone (**Figure 4J**). This conclusion was validated at multiple time points, including 3, 5, and 10 years.

### The correlation of risk score and immune cell infiltration, predicted immunotherapy response, and ICIs

**Figure 5:**
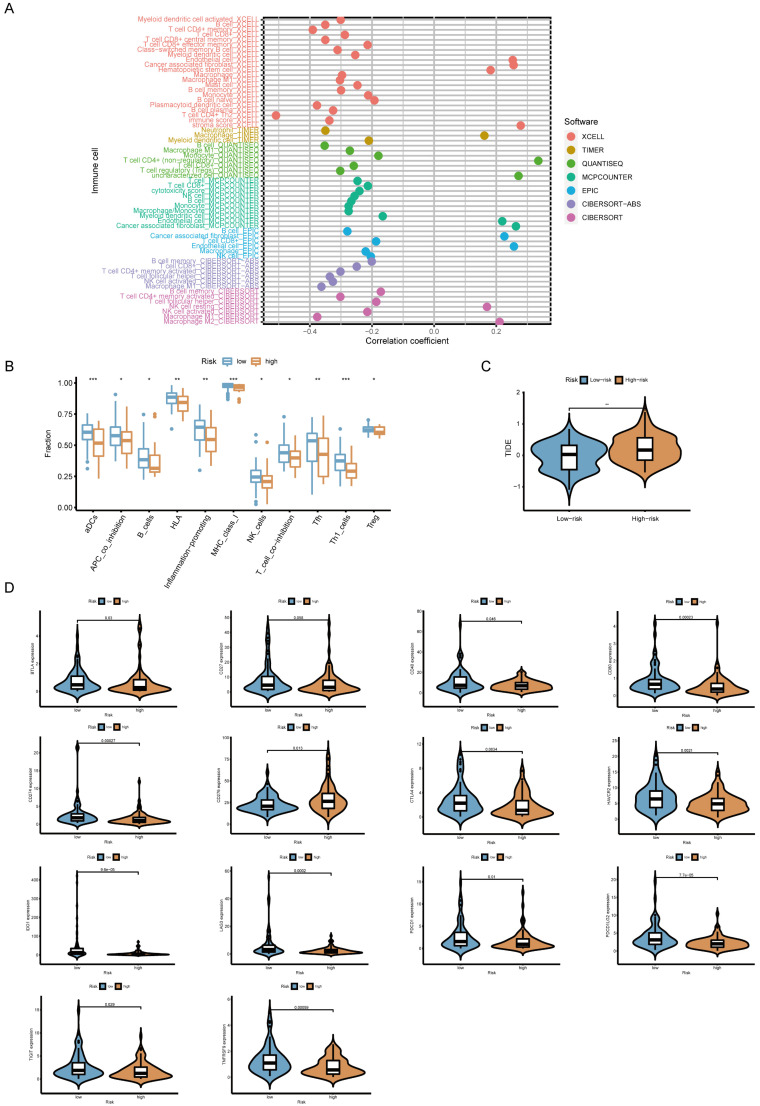
** Association between risk score and immune characteristics. (A)** Lollipop plot showing correlations between risk score and tumor-infiltrating immune cells.** (B)** Comparison of immune cell function between high-risk and low-risk groups.** (C)** Predicted response to immune checkpoint inhibitors (ICIs) in high- and low-risk groups based on TIDE score analysis.** (D)** Violin plots illustrating expression levels of immune checkpoint-related genes in different risk groups.

Whether the prediction model was related to the tumor immune microenvironment needs to be further investigated. We evaluated the immune infiltration status among the samples using XCELL, TIMER, QUANTISEQ, MCPCOUNTER, EPIC, CIBERSORT-ABS, and CIBERSORT algorithms. A high-risk score was positively correlated with M2 macrophage, cancer-associated fibroblast infiltration, stromal score, endothelial cells and hematopoietic stem cells (**Figure 5A**). Meanwhile, it was negatively associated with the level of most immune infiltrating cells and their corresponding immune function (**Figure 5B**). TIDE score is a good predictor of the efficacy of anti-PD1 and anti-CTLA4 therapy that reflects tumor immune dysfunction and exclusion in patients 26. To assess potential responses to immune checkpoint inhibitor therapy, we analyzed the expression of key immune checkpoints (PD-L1, PD-1, CTLA-4, LAG3, IDO1). High-risk patients showed lower expression of multiple ICIs and higher TIDE scores, suggesting reduced sensitivity to immunotherapy (**Figure 5C, D**).

### Risk score correlates with TMB and chemotherapy sensitivity

**Figure 6:**
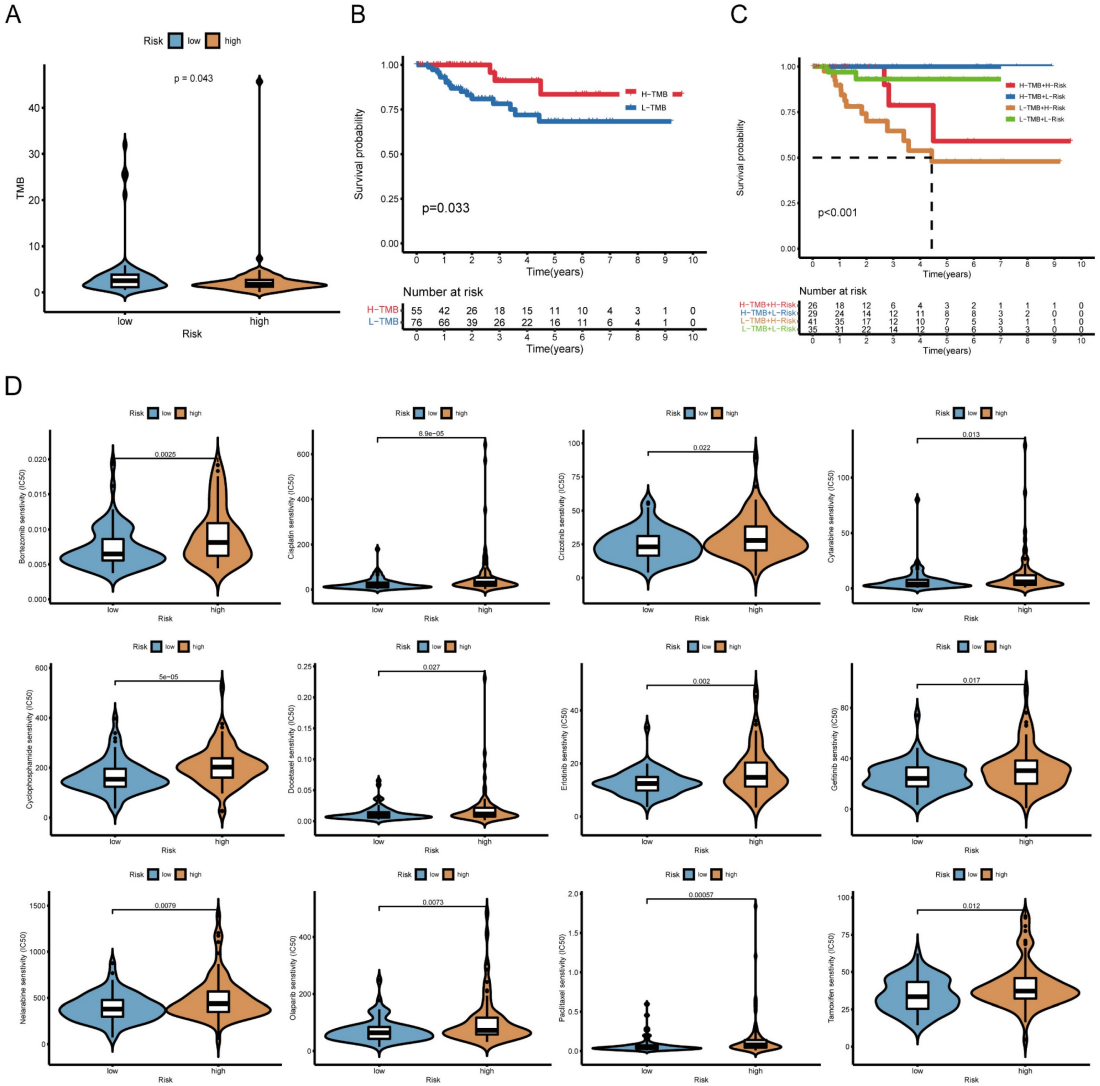
** Relationship between the risk model, tumor mutational burden (TMB), and chemotherapy response. (A)** Boxplot comparing TMB levels between high-risk and low-risk groups. **(B)** Kaplan-Meier survival curves for high-TMB and low-TMB patients. **(C)** Prognostic stratification based on a combination of risk score and TMB. **(D)** Boxplots depicting the correlation between risk score and IC50 values of 12 commonly used chemotherapeutic drugs (another 38 drugs are presented in **Supplementary Figure 3**).

Further analysis revealed that high-risk patients exhibited lower TMB (**Figure 6A**). Survival analysis showed that low-TMB patients had worse overall survival, and combined TMB-risk stratification identified patients with the poorest prognosis (**Figure 6B, C**). Moreover, the waterfall plot ranks the frequency of gene mutations in low-risk and high-risk patients (**Supplementary Figure 2**).

We also examined whether the risk score could predict chemotherapy response. High-risk patients had higher IC_50_ values for multiple chemotherapeutic agents, including bortezomib, cisplatin, cytarabine, cyclophosphamide, docetaxel and paclitaxel, suggesting lower drug sensitivity (**Figure 6D**). Information on the sensitivity to another 38 drugs, including not only chemotherapy drugs but also inhibitors of various carcinogenic pathways and enzymes, is listed in **Supplementary Figure 3**. Gene set variation analysis (GSVA) further indicated that high-risk group was enriched for drug metabolism cytochrome P450 and ECM receptor interaction, whereas low-risk group exhibited enrichment in immune-related pathways (**Supplementary Figure 4**).

### Functional validation of hub lncRNA in TNBC

**Figure 7:**
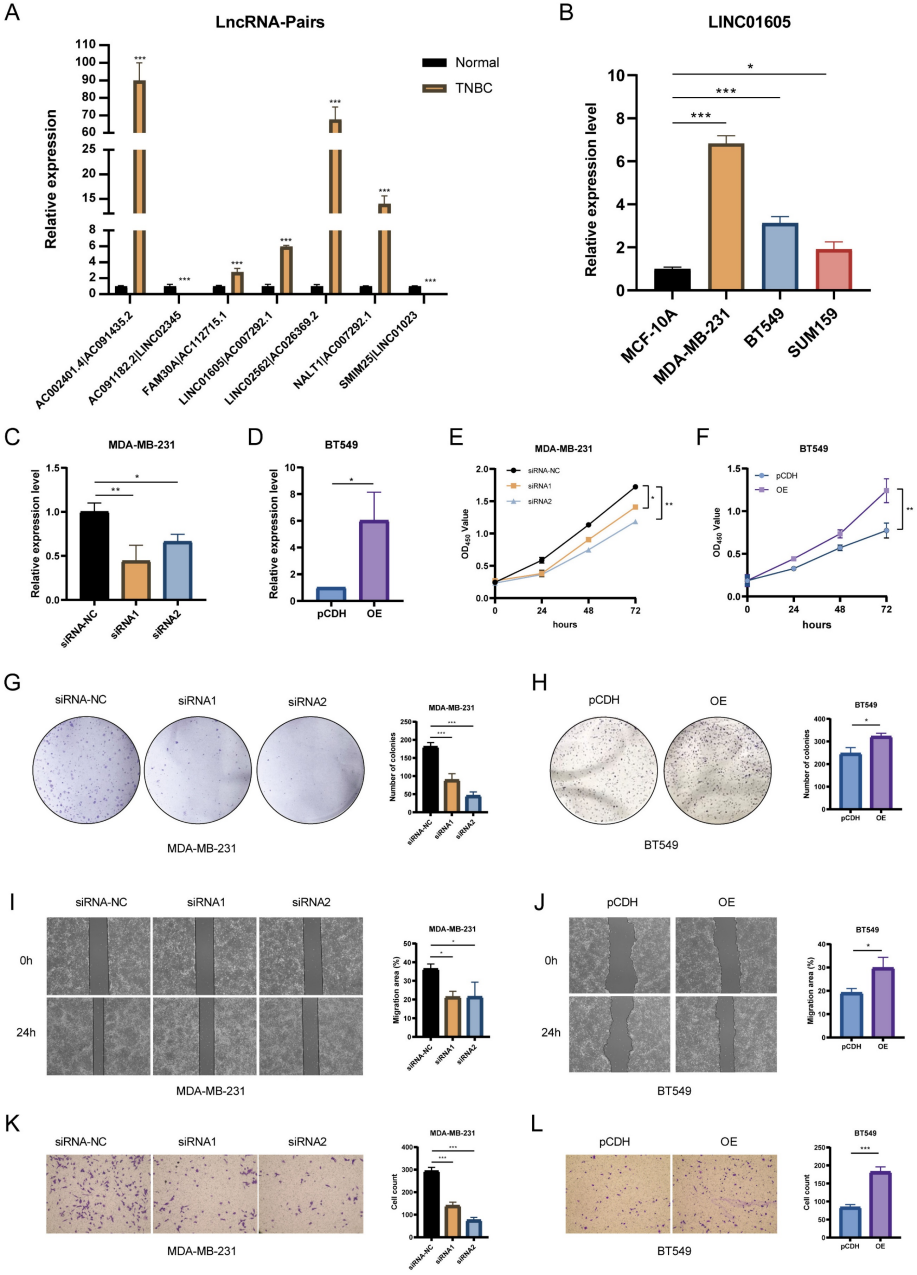
** Functional validation of LINC01605 in TNBC. (A)** RT-qPCR analysis showing differential expression of key lncRNA pairs in TNBC tissues. **(B)** LINC01605 expression levels in TNBC cell lines (MDA-MB-231, BT549, SUM159) and normal mammary epithelial cells (MCF10A). **(C, D)** RT-qPCR confirming LINC01605 knockdown efficiency in MDA-MB-231 and overexpression efficiency in BT549 cells. **(E, F)** CCK-8 assay demonstrating the effect of LINC01605 on cell viability. **(G, H)** Colony formation assay showing the impact of LINC01605 on TNBC cell proliferation. **(I, J)** Wound healing assay assessing the role of LINC01605 in cell migration (scale bar: 200 µm). **(K, L)** Transwell assay evaluating the effect of LINC01605 on cell invasion (scale bar: 50 µm). * *p* < 0.05, **p < 0.01, ***p < 0.001.

The expression levels of lncRNA pairs in TNBC tissues and adjacent normal breast tissues were quantified using RT-qPCR. The results revealed that five key lncRNA pairs (AC002401.4|AC091435.2, FAM30A|AC112715.1, LINC01605|AC007292.1, LINC02562|AC026369.2, and NALT1|AC007292.1) were upregulated, while two pairs (AC091182.2|LINC02345 and SMIM25|LINC01023) were downregulated (**Figure 7A**). To further explore the biological significance of these lncRNAs, we selected LINC01605, which demonstrated the most significant prognostic impact, for experimental validation. RT-qPCR analysis confirmed that LINC01605 expression was significantly elevated in human TNBC cell lines compared to MCF10A (normal mammary epithelial cells) (**Figure 7B**). Additionally, RT-qPCR was used to assess the efficiency of LINC01605 knockdown (**Figure 7C**) and overexpression (**Figure 7D**) in MDA-MB-231 and BT549 cells. Functional assays revealed that LINC01605 knockdown significantly inhibited cell proliferation (CCK-8 and colony formation assays), migration (wound healing assay), and invasion (transwell assay). Conversely, LINC01605 overexpression promoted these cellular processes (**Figure 7E-L**). These findings suggest that LINC01605 plays a crucial role in TNBC progression and may serve as a potential therapeutic target.

## Discussion

TNBC remains a major clinical challenge due to its aggressive nature, high recurrence rate, and limited targeted treatment options. The lack of hormone receptors and HER2 expression makes conventional endocrine or targeted therapies ineffective, leaving chemotherapy as the primary treatment. However, tumor recurrence continues to pose a significant obstacle, necessitating the development of novel prognostic models and therapeutic strategies 12, 27.

Compared to other breast cancer subtypes, TNBC exhibits higher levels of tumor-infiltrating lymphocytes (TILs), PD-L1 expression, and TMB, making it a promising candidate for immune checkpoint inhibitor therapy 28. Nonetheless, the heterogeneous nature of TNBC means that not all patients respond favorably to immunotherapy, emphasizing the need for biomarkers that can predict treatment response and recurrence risk 29.

In this study, we developed a lncRNA-based risk model utilizing a refined cyclical single pairing method and a binary matrix, which allowed for the identification of lncRNA pairs differentially expressed in cancerous and normal tissues.In this study, we developed a lncRNA-based risk model utilizing a refined cyclical single pairing method and a binary matrix, which allowed for the identification of lncRNA pairs differentially expressed in cancerous and normal tissues. Unlike traditional approaches that rely on absolute expression levels, our method reduces variability introduced by factors such as regional differences, racial backgrounds, and platform inconsistencies 22. Our risk model offers significant potential for clinical translation in TNBC management. The risk score can guide adjuvant therapy decision-making by identifying high-risk patients who may benefit from intensified treatment regimens, such as combination chemotherapy or novel targeted therapies, thereby optimizing therapeutic outcomes. Furthermore, integrating lncRNA-based signatures into liquid biopsy platforms could enable non-invasive monitoring of disease progression and treatment response, facilitating early detection of recurrence and personalized treatment adjustments 30. These applications highlight the model's utility in advancing precision medicine for TNBC patients.

Although our experimental results confirm the oncogenic role of LINC01605 in promoting TNBC cell proliferation, migration, and invasion, the precise molecular mechanism remains to be elucidated. Recent studies in nasopharyngeal carcinoma have shown that LINC01605 can activate the NF-κB pathway, forming a positive feedback loop that enhances tumor proliferation and survival 31. Since NF-κB signaling is known to facilitate immune evasion by promoting pro-inflammatory cytokine release, PD-L1 expression and T cell exclusion 32. Furthermore, lncRNAs have been reported to regulate antigen presentation and checkpoint pathways such as PD-1/PD-L1, implying a potential role for LINC01605 in shaping the immunosuppressive tumor microenvironment 33. These mechanisms may underlie the observed negative correlation between LINC01605 expression and immune infiltration in our model, warranting further investigation into LINC01605-mediated modulation of immune escape in TNBC.

A critical factor influencing TNBC prognosis is the interaction between tumor-infiltrating immune cells and immune checkpoints, which plays a crucial role in determining the efficacy of immune checkpoint blockade therapy 34. Our findings revealed a negative correlation between the risk score and immune cell infiltration, suggesting that high-risk patients may exhibit immune evasion and reduced sensitivity to ICIs (*p* < 0.05). Moreover, TIDE score analysis further indicated that high-risk patients were more prone to immune escape mechanisms, supporting the clinical relevance of our model in predicting immunotherapy outcomes. Given that key immune checkpoints such as PDCD1 (PD-1), CTLA-4, LAG3, and IDO1 act as negative immune regulators, their lower expression in high-risk patients implies a diminished response to immune checkpoint blockade therapy 35-38. Although some ICIs, such as atezolizumab and pembrolizumab, have been approved for TNBC treatment, chemotherapy remains the mainstay therapy for both early-stage and advanced TNBC 39. Our study demonstrated that risk scores were significantly associated with chemotherapeutic drug sensitivity, highlighting the potential utility of this model in guiding personalized treatment selection.

Emerging research has revealed additional dimensions in the interplay between lncRNAs and tumor immunity. Certain lncRNAs can modulate antigen presentation by influencing MHC class I/II molecule expression or interfering with antigen processing pathways, thereby shaping immune recognition 40. Others have been implicated in promoting immune resistance by regulating checkpoint molecules (e.g., PD-L1) or suppressing effector T cell infiltration 41. Moreover, the advent of spatial transcriptomics and single-cell multi-omics now enables the mapping of lncRNA expression in relation to specific immune cell niches within the tumor microenvironment 42, 43. These technologies hold promise for identifying spatially restricted lncRNA-immune interactions that contribute to immune evasion, and may uncover novel therapeutic targets. As the field evolves, integrating spatial and functional information will be crucial to unravel the complex roles of lncRNAs in immune modulation and resistance to immunotherapy.

Despite the promising results, this study has several limitations. (1) our model was developed and validated using retrospective transcriptomic data from the TCGA and GEO datasets, but external validation across multiple, prospective, and multi-center cohorts was not conducted. Future studies are needed to assess the model's generalizability in broader clinical settings. (2) although LINC01605 was experimentally validated as a potential oncogenic lncRNA in TNBC, the molecular mechanisms by which it regulates immune evasion and tumor progression remain to be fully elucidated. Investigation into associated RNA-binding proteins, downstream signaling pathways, and epigenetic modifications will be essential for mechanistic understanding. (3) while our model incorporates lncRNA pairs to improve robustness, it does not integrate other layers of biological regulation such as somatic mutations, methylation, proteomics, or spatial transcriptomics, which may further enhance its predictive performance. (4) only one lncRNA from the model (LINC01605) was functionally validated; the roles of other lncRNAs included in the risk signature remain to be verified in future studies. (5) our cohorts did not include patients treated with ICIs, limiting the direct clinical applicability of our model in the context of immunotherapy. Incorporating ICI-treated cohorts in future validation efforts will be important to confirm the model's utility for guiding immunotherapy decisions.

## Conclusion

In summary, we developed a novel relapse- and immune-related lncRNA pair-based model that effectively predicts tumor recurrence, prognosis, and immunotherapy response in TNBC. The nomogram constructed using the risk score provides an innovative tool for patient stratification, aiding in the identification of those who may benefit from immunotherapy and chemotherapy. Furthermore, our study highlights LINC01605 as a promising target for therapeutic intervention. Future investigations should aim to validate these findings in larger patient cohorts and explore the mechanistic role of LINC01605 in TNBC progression.

## Supplementary Material

Supplementary figures and tables.
