# Supplementary material for: Dual-Function RNA Biomarkers: Integrating Relapse Prediction and Immune Profiling in Triple-Negative Breast Cancer
Source: Int J Med Sci. 2025 Aug 11;22(14):3763–78. doi: 10.7150/ijms.119142 (PMC12434821; doi:10.7150/ijms.119142)
Supplement: Supplementary file 1 — Supplementary figures and tables. [file ijmsv22p3763s1.pdf]

Supplementary Figures

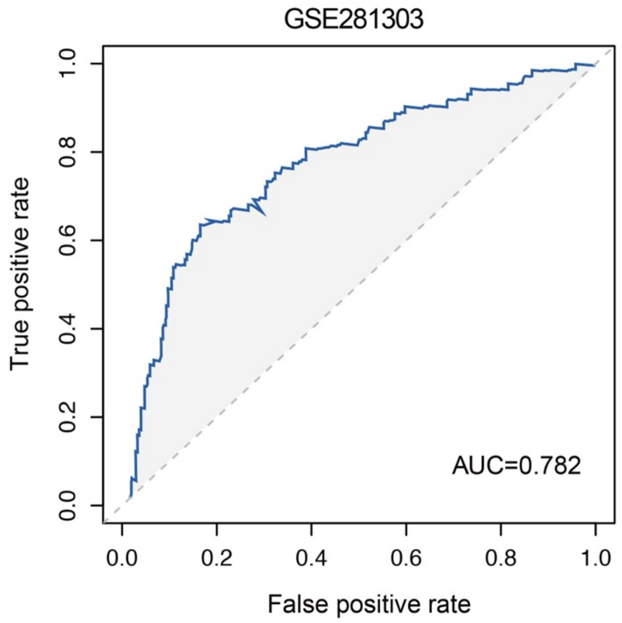

Supplementary Figure 1. The receiver operating characteristic curve for evaluating the stability of the prediction model in GSE281303 external cohort.

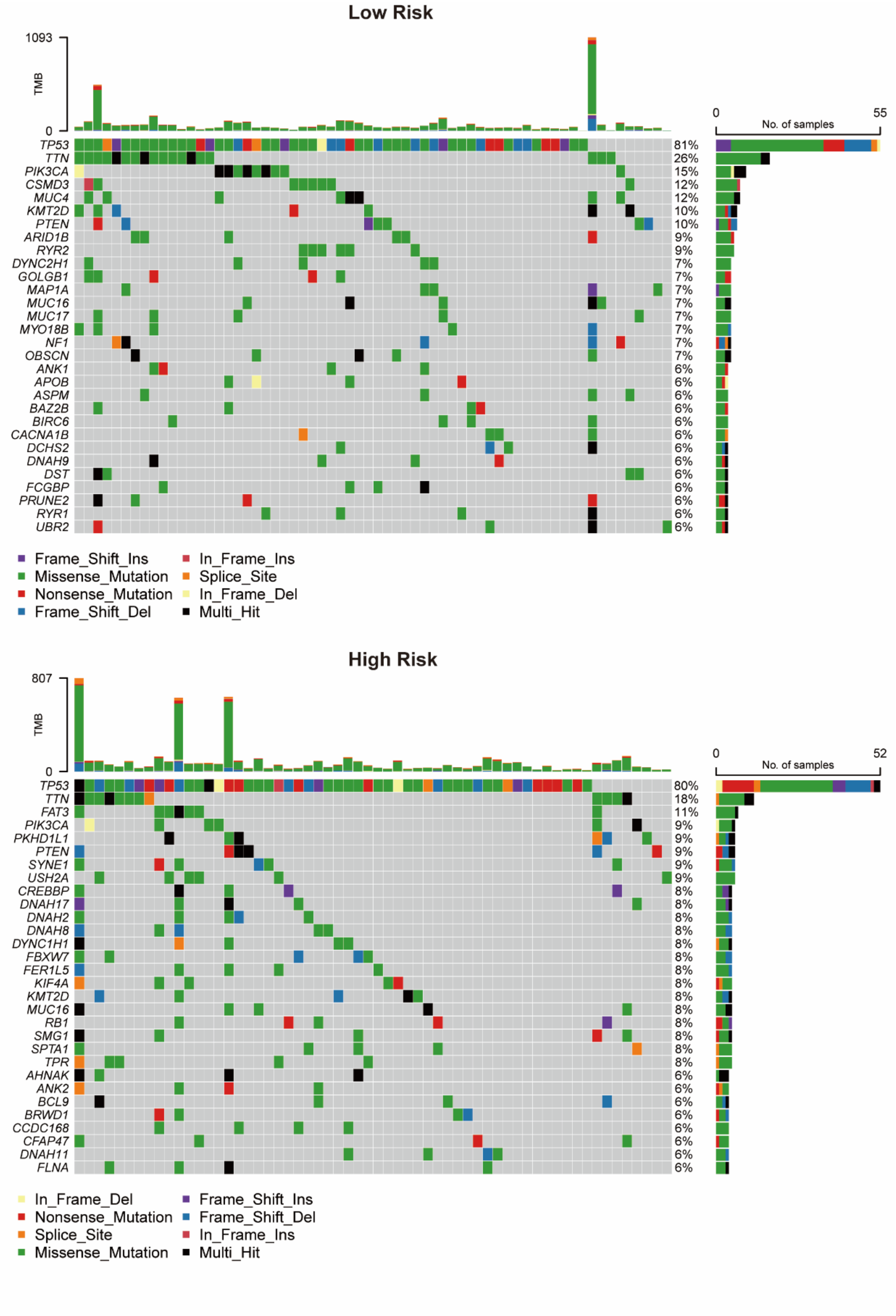

Supplementary Figure 2. The waterfall plot shows gene mutations in low-risk and high-risk patients ranked in order of frequency.

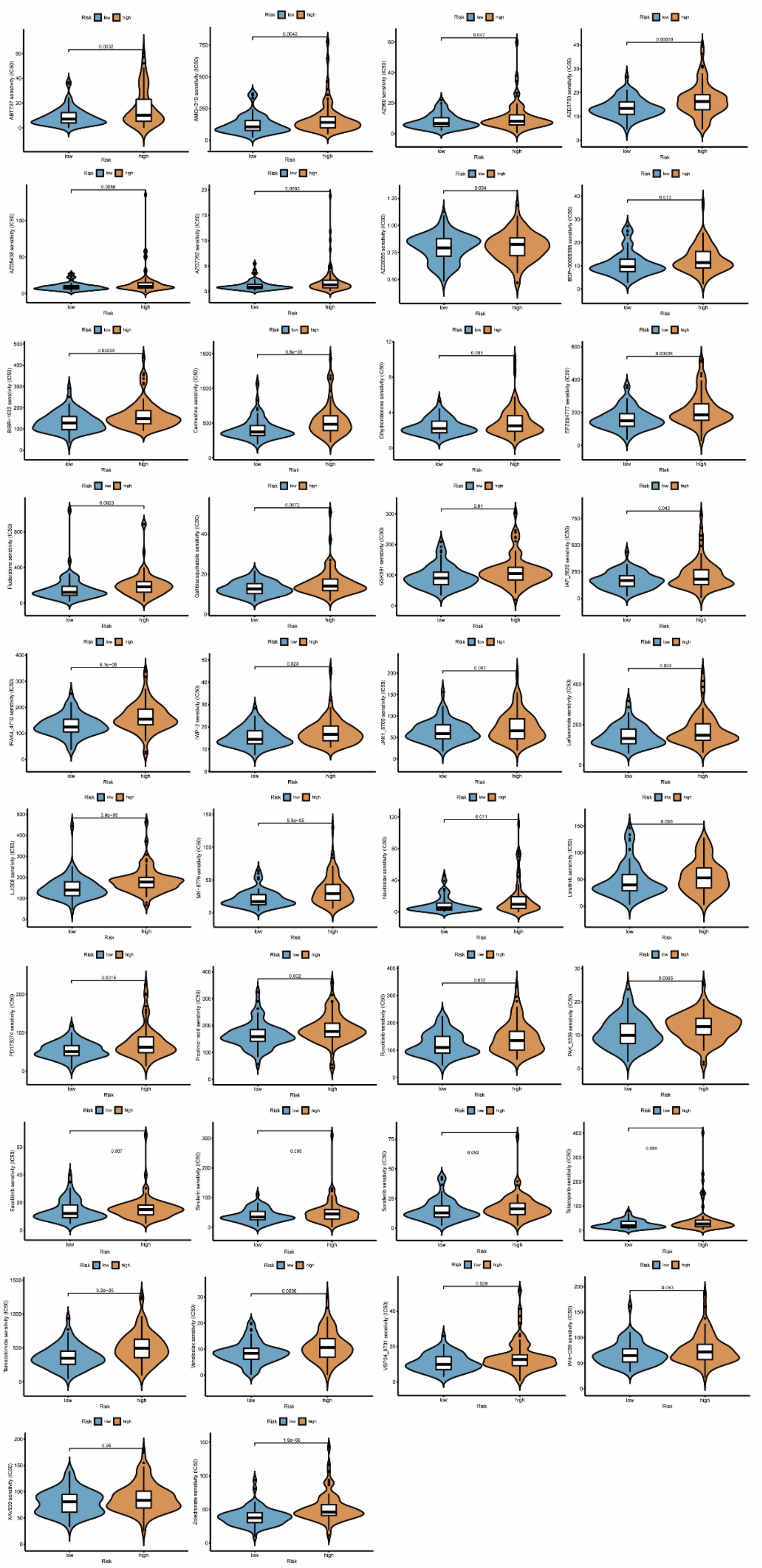

Supplementary Figure 3. The relationship between the risk score and the efficacy of commonly administered tumor therapeutic drugs (the other 38 drugs).

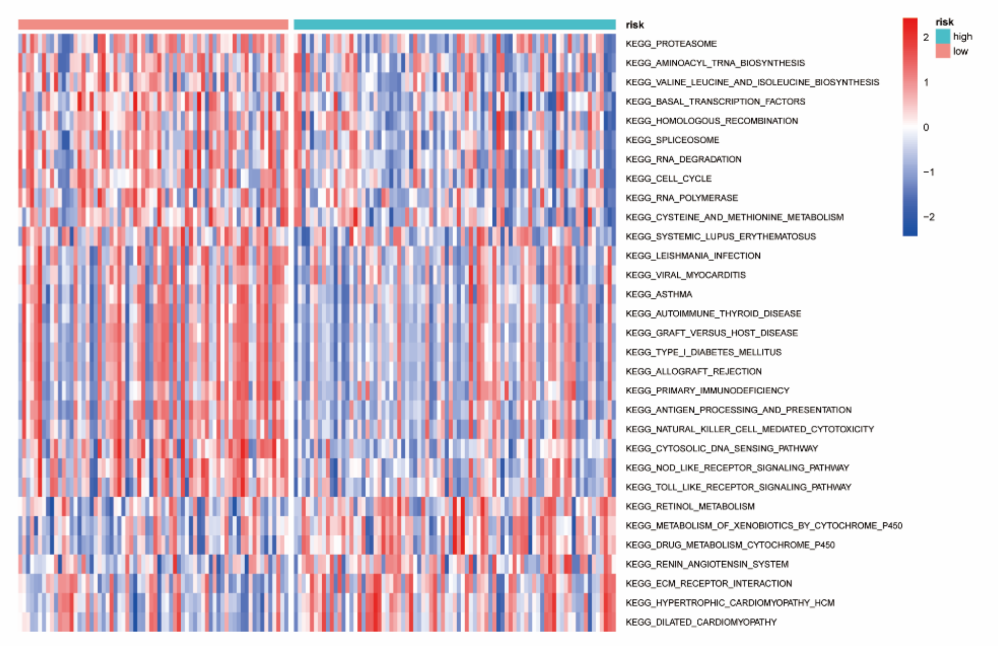

Supplementary Figure 4. Gene set variation analysis (GSVA) between the high- and low-risk subgroups.

Supplementary Tables

Supplementary Table 1. Primers for 13 lncRNAs involved in the model and U6 as an endogenous reference.

Genes	FORWARD	REVERSE
AC002401.4	TCCTGTCTGTGTTCCCTGTTCCC	CCCGCCACTTTGATGCTTTCTTTG
AC007292.1	CCGTGGAGTTGGAGGTCATTGTG	AAGTGCTGGGATTAAAGGCGTGAG
AC026369.2	GAAAACCAGGAGAGCATGGAGCT	TTTTTTCGCGTTCATTATCTCAT
AC091182.2	AAAGACAAGGCGAGGACAAGAAGAG	TTCGCTACCCACCAAACCCATTTC
AC091435.2	CTGGCTGCTGCTTTGCTATTTGTG	GTCCTGGTGATCCTTATGCGTGATG
AC112715.1	AGCGGGAAGACCTGAACCTTGG	GTGGAATGGCAGGAAGAGCAGAG
FAM30A	TACCTCTCAGCCTCCAGCGTTG	CTCTTGCTCTTGCTCACTCACACTC
LINC01023	GAACAGACACTCACGGACACATGG	TCCAGGGCTCAGCTCACAGAAG
LINC01605	CAACTCATTCCCGTTACAAACA	CATCTCAACTGCCTCTGTCTCC
LINC02345	TGGTCTTGCTTGGCTTTCTGGATG	TTTAATGCCTGGCGGCTGTAGC
LINC02562	TCTCTGGGTGGAATGTCAC	TTTACTGGGCACTTGTCCT
NALT1	GTCATCCAGTAGGCTCAAG	ATAAGTGGAGAAAGGCAGAT
SMIM25	AGCACAGAGAAGTTCAGTGATGGAC	AGGCAGTGTAGCATAGTGGGTAGG
GAPDH	CAAATTCCATGGCACCGTCA	GACTCCACGACGTACTCAGC

Supplementary Table 2. Twenty-five lncRNA pairs with prognostic significance were identified by univariate Cox analysis. HR, Hazard Ratio.

LncRNA pairs	HR	HR.95L	HR.95H	P.value
AC091182.2|LINC02345	5.064138129	1.735017	14.78112	0.002996
LINC00924|CHRM3-AS2	4.749769442	1.780373	12.67168	0.001858
LINC01605|LINC02528	4.635450001	1.728771	12.42929	0.002306
LINC01605|LINC01914	4.444390801	1.646676	11.99544	0.003235
LINC02568|LINC02446	4.268813567	1.943966	9.374018	0.000299
AC091182.2|MIR223HG	4.025997943	1.780728	9.102267	0.000819
LINC02562|AL512306.2	3.99948367	1.771158	9.031307	0.000852
LINC00924|LINC02528	3.925490551	1.731067	8.901722	0.001062
LINC02568|AL356215.1	3.686212849	1.670708	8.13318	0.001233
LINC01605|AC007292.1	3.636399438	1.650008	8.014141	0.001365
LINC02568|AC004921.1	3.315824215	1.512068	7.271292	0.002771
LINC02562|AC007292.1	3.294242973	1.463281	7.416235	0.003984
LINC02562|AC026369.2	3.294242973	1.463281	7.416235	0.003984
NALT1|LINC02446	3.272629991	1.444307	7.415396	0.004499
LINC00924|FAM30A	3.263601046	1.472744	7.232142	0.003574
NALT1|AC007292.1	3.245370971	1.471902	7.15566	0.003521
LINC00924|LINC02771	3.199159934	1.458776	7.015897	0.003703
AC002401.4|AC091435.2	3.186179679	1.439818	7.050713	0.004244
LINC02568|LINC00926	3.108980442	1.409174	6.859166	0.004961
SMIM25|LINC01023	0.302628583	0.133639	0.685308	0.004156
AL662844.4|LINC02562	0.295373521	0.130061	0.670806	0.003568
AL512353.1|LINC02562	0.276743126	0.122106	0.627217	0.002088
LINC02528|AC096921.2	0.189003161	0.064695	0.552162	0.002321
FAM30A|AC112715.1	0.182929573	0.062609	0.534478	0.001902
LINC02528|AC112715.1	0.112634529	0.038358	0.330743	7.09E-05

Supplementary Table 3. Seven lncRNA pairs were used to establish the risk assessment model. HR, Hazard Ratio.

LncRNA Pairs	Coef	HR	HR.95L	HR.95H	P.value
AC002401.4|AC091435.2	0.874926	2.398697	1.037407	5.546275	0.040772
AC091182.2|LINC02345	1.373479	3.949065	1.314962	11.85975	0.014366
FAM30A|AC112715.1	-1.83276	0.159971	0.051696	0.495019	0.001473
LINC01605|AC007292.1	1.883262	6.574917	2.281041	18.95167	0.000489
LINC02562|AC026369.2	1.208039	3.346915	1.402983	7.9843	0.006464
NALT1|AC007292.1	1.260132	3.525889	1.479607	8.402155	0.004452
SMIM25|LINC01023	-1.84595	0.157875	0.050915	0.489535	0.001388
